# Optimization of rGO-PEI/Naph-SH/AgNWs/Frt/GOx nanocomposite anode for biofuel cell applications

**DOI:** 10.1038/s41598-020-65712-8

**Published:** 2020-06-02

**Authors:** Inamuddin, Nimra Shakeel

**Affiliations:** 1 Chemistry Department, Faculty of Science, King Abdulaziz University, Jeddah, 21589 Saudi Arabia; 2 Advanced Functional Materials Laboratory, Department of Applied Chemistry, Faculty of Engineering and Technology, Aligarh Muslim University, Aligarh, 202 002 India; 3 Department of Chemistry, Faculty of Science, Aligarh Muslim University, Aligarh, 202002 India

**Keywords:** Chemistry, Energy science and technology

## Abstract

The present study reports a new nanocomposite design using surface modified silver nanowires decorated on the surface of polyethyleneimine (PEI), a cationic polymer acting as glue for anchoring nanowires and reduced graphene oxide (rGO). The synthesized nanocomposite was employed as a promising electrode material for immobilization of biomolecules and effective transportation of electron, in enzymatic biofuel cell (EBFCs) application. The synthesized nanocomposite was confirmed by analytical techniques, for instance, Fourier transform infrared spectroscopy (FTIR), scanning electron microscopy (SEM), transmission electron microscopy (TEM). The electrochemical behaviour of the nanobioelectrocatalysts rGO-PEI/Frt/GOx, rGO-PEI/AgNWs/Frt/GOx, and rGO-PEI/Naph-SH/AgNWs/Frt/GOx was determined by cyclic voltammetry (CV), electrochemical impedance spectroscopy (EIS), and linear sweep voltammetry (LSV). The maximum current density obtained by the modified bioanode was found to be 19.9 mA cm^−2^ at the limiting glucose concentration of 50 mM in PBS (pH 7.0) as supporting electrolyte at a scan rate of 100 mVs^−1^.

## Introduction

Nanoscience and nanotechnology are gaining increasing interest due to their ability to tailor diverse nanoscale materials. Interestingly, nanomaterials possess excellent properties such as large surface to volume ratio, high surface reactivity, and different morphologies^1–3^. These dynamic properties of nanomaterials make them advantageous in any conceivable domain like in the food industry^4^, electronics^5^, pharmaceuticals^6^ and agriculture^7^. Nanoscale materials have also been utilized in enzymatic biofuel cells (EBFCs) to improve their performance^8^. EBFC is one of the reliable energy generation technologies for powering miniaturized bioelectronic systems. Enzymatic biofuel cells relay on two separate redox reactions coupled with the enzyme functionalized electrode materials linked through an external circuit^9^. The potential chemical energy stored in biomolecules is converted into electrical energy by a suitable biocatalyst. The merits of the generation of electrical power via EBFCs are their eco-friendly and feasible operational conditions, use of renewable physiological fluids (e.g., glucose^10^, fructose^11^, etc.) and specificity of biocatalysts. For example, the glucose oxidase (GOx)^12^ is a bioelectrocatalyst that specifically catalyzes the most exploited glucose fuel, which is ubiquitous in the living systems. However, the redox-active centre of GOx is deeply seated inside its protein shell which makes the electron transfer difficult through it^13^. Two main strategies such as the use of redox active mediator and orientated immobilization of enzymes have been used to mitigate this problem^14–16^. Furthermore, the capability of nanomaterials such as carbon nanotubes or metal nanoparticles to link directly to the electroactive enzyme is of immense interest to facilitate the direct electron transfer (DET)^17^ while in the case of mediated electron transfer (MET), a mediator is employed in between the electrode surface and the enzyme that acts as an electron relay center by reducing the path length for electron transfer^14^. Reportedly, a 14-fold increase in catalytic current was obtained by using a free naphthoquinone to a glucose oxidase (GOx)/multi-walled carbon nanotube (MWCNT)-based anode^16^. Nowadays, ferritin (Frt) has been employed as an active redox mediator that assists the electron shuttling between redox active site of GOx and the electrode surface^18^. The biocompatible nature of Frt makes it more favourable than the other toxic mediators which enhances its utilization in a glucose-based biofuel cell^19^. The specificity of the GOx towards the oxidation of glucose leads to the development of the miniaturized membrane-less system^20^ which makes EBFCs an essential tool in the field of implantable power sources, self-powered sensors^21^, and portable electronic devices^22^. However, despite all the promises, still, there are specific roadblocks such as low power output and short life span which limit their real-world applications.

Over the past few decades, the development of advanced supporting materials is the intellectual strategy in material research to achieve efficient immobilization of enzymes^23^. Recent progress in nanoscience and a detailed understanding of the chemistry of nanomaterials ensure cost-efficient development of nanobiocatalysts^24^. To date, carbon nanomaterials (e.g., carbon nanotubes, graphene, etc.) have been exploited as a versatile supporting material for the enzyme wiring^25^. Currently, researchers are showing great interest in exploiting the intrinsic properties of 2D graphene and its derivatives (graphene oxide, reduced graphene oxide) in various fields such as nanoelectronics^26^, drug delivery^27^, environmental remediation^28^ and designing electrode materials^29^, on account of their high mechanical strength, good electrical conductivity, free electron mobility, high aspect ratio, flexible tunability, and lightweight. The high surface to volume ratio of rGO offers the large accessible surface area for enzyme loading^30^. To date, various combinations of rGO with conducting polymers such as PANI-rGO^31^, polypyrrole-rGO^32^ have been reported as potential electrode materials. However, very few articles have been published on polyethyleneimine (PEI) with carbon nanomaterials^33^ in biofuel cells. However, some applications have been explored in biosensors^34^, filtration membrane^35^ and drug delivery^36^. PEI is a biocompatible porous cationic polymer that is easily prepared and economically feasible. It is also renowned for its excellent enzyme immobilizing ability. Under normal conditions, it possesses a positive charge which maintains strong interaction with GOx. Although PEI possesses all these fascinating attributes, still its conductivity is lowe as compared to that of others conducting polymers such as polypyrrole, polythiophene, and polyaniline which limits its direct application as electrode materials^37^. To address the above issues, the use of metal nanoparticles (MNPs) can be a promising strategy to improve the conductivity of PEI. The utilization of surface modified MNPs overcomes the problem of their aggregation and simultaneously promotes their interaction with the polymer matrix. A variety of MNPs such as gold^38^, magnetite^39^ and silver nanoparticles^40^ have been explored for electrodes modification due to their high catalytic activity. Similarly, the capability of electron transfer surges by introducing the thiol group on the surface of MNPs^41^. Furthermore, the morphology of MNPs, their shape, size, and concentration influence the properties of the nanocomposite. Besides, one-dimensional nanowire has high surface activity due to the very high specific surface area which tremendously enhances the loading capability of the nanocomposite.

Herein, a nanocomposite composed of silver nanowires (AgNWs) modified with naphtalenethiol coupling agent decorated on the surface of cationic polymer polyethyleneimine (PEI) and the rGO is prepared. Here, PEI acts as glue for anchoring nanowires assisted by thiol linkage of naphthalene thiol. Apart from it, PEI/AgNWs@Naph-SH acts as a sandwich between rGO sheet and GOx in the developed biocatalyst rGO/PEI/AgNWs@Naph-SH/Frt/GOx. Moreover, modification of AgNWs with a napthalenethiol stabilizing agent makes a protecting covering around the NWs that prevents them from further ionization. The contribution of each component of the synthesized nanocomposite rGO/PEI/AgNWs@Naph-SH plays a significant role in improving the stability of the bioanode by establishing (i) hydrophobic interaction between GOx and the PEI/AgNWs@Naph-SH nanocomposite and (ii) π conjugation between GOx and naphthalenethiol@AgNWs that assist the electron transfer process. In this study, three different types of electrodes; rGO/PEI/Frt/GOx, rGO/PEI/AgNWs/Frt/GOx, and rGO/PEI/AgNWs@Napth-SH/Frt/GOx have been developed and compared to confirm the contribution of each component in enzyme wiring.

## Materials and Methods

The polyethyleneimine (PEI) (average molecular weight 800 by light scattering), reduced graphene oxide (rGO), napthalenethiol (Naph-SH,99%), silver nitrate (AgNO_3_), ferritin (10 mg mL^−1^ in 0.15 M NaCl), glucose oxidase (GOx) from Aspergillus niger and 2% glutaraldehyde aqueous solution were purchased from the Sigma-Aldrich, India. Sodium chloride (NaCl), potassium bromide (KBr), phosphate buffer saline (PBS, pH 7.0), polyvinylpyrrolidone (PVP, molecular weight 1,300,000) and ethylene glycol (EG) were procured from Alfa Aesar, India.

### Instruments used

The Fourier transform infrared spectroscopy (FTIR) was carried out with the help of Nicolet iS50 FT-IR instrument operating in the range of 4000–400 cm^−1^, and X-ray diffraction (XRD) analysis was performed by using Rigaku Smart Lab X-ray diffractometer, to elucidate the functional groups and the crystalline structure of the nanocomposite, respectively. The morphologies of the synthesized nanocomposites were examined using scanning electron microscopy (SEM) (JSM, 6510 LV, JEOL, Japan) and transmission electron microscopy (TEM) (TEM 2100, JEOL, Japan) operated at 200 kV on a carbon-coated copper grid. Moreover, the electrochemical behaviour of the fabricated bioanode was investigated by a three-electrode assembly having glassy carbon electrode as working, a Pt wire as a counter and Ag/AgCl (3 M KCl) as a reference electrode, coupled with the potentiostat/galvanostat (PGSTAT 302 Autolab, Switzerland).

### Synthesis of silver nanowires (AgNWs)

The synthesis of silver nanowires (AgNWs) through controlled morphology can be obtained by keeping the molar ratio of [PVP] to [AgNO_3_] as mentioned elsewhere^42^. Briefly, PVP (75 mg) was dissolved in 10 mL of EG with continuous heating and magnetic stirring (550 revolutions per minute) of the solution in an oil bath for 60 minutes at 160 °C temperature. After that, 20 µL of KBr (1.8 mM) and 40 µL of NaCl (3.5 mM) solutions prepared in EG were added into the above solution followed by stirring and heating of the mixture for 30 minutes. Then, 5.2 ml AgNO_3_ solution was pipetted out and continuously added to the above mixture while maintaining the flow rate of 0.2 mL min^−1^. Following this, the solution was refluxed at 160 °C upto 3 h and the noticeable change in the colour was observed from yellow to turbid grey which indicates the formation of AgNWs. After cooling, the solution was agitated for 10 minutes in an ultra-sonication bath and centrifuged at 7000 rpm. Lastly, final pure AgNWs as shown in the SEM micrograph were obtained by decanting the supernatant liquid.

### Synthesis of rGO/PEI/AgNWs@Naph-SH nanocomposite

Firstly, the 100 mg of rGO was dispersed in 5 mL double distilled water (DDW) for the proper dispersion of rGO sheets and then 20 mL PEI polymer was added into it; the negatively charged rGO sheets interact with positively charged PEI resulting in the homogenous suspension. Simultaneously, in a separate beaker, 1 mL of already synthesized AgNWs were ultrasonicated with 2 mg naphthalenthiol coupling agent and centrifuged for 7 minutes. This resulting mixture was added into above mentioned rGO/PEI homogenous suspension and diluted it with distilled water so that the final concentration of the solution reached 2.5 mg mL^−1^. The mixture was ultrasonicated for 10 minutes allowing the reaction to complete. The supernatant was dumped followed by 7 minutes centrifugation and this step was repeated three times by washing with DDW to ensure the removal of excessive PEI. The same procedure was followed for the synthesis of rGO/PEI/AgNWs without the modification of AgNWs^41^.

### Preparation of rGO/PEI/AgNWs@Naph-SH nanocomposite dispersion

The consistency of the synthesized (rGO/PEI/AgNWs@Naph-SH) nanocomposite was adjusted by adding double-distilled water into it and the resultant dispersion was used as catalytic ink.

### Fabrication of bioanode

The bioanodes rGO/PEI/Frt/GOx, rGO/PEI/AgNWs/Frt/GOx, and rGO/PEI/AgNWs@Naph-SH /Frt/GOx were developed by the drop-casting method. Before using, a 3 mm diameter glassy carbon electrode (GCE) was cleaned as described elsewhere^43^. For designing the bioanode, firstly 6 µL of the synthesized nanocomposite (rGO/PEI/AgNWs@Naph-SH) dispersion was drop-cast on the top of GCE by spreading evenly using micro-pipette and letting it dry for 5–6 hours at room temperature. After that, 5 µL of Frt solution was applied on the dried GCE and allowed to dry for 30 minutes. Next, 6 µL of GOx solution was loaded on the modified GCE. Thereafter, 1.5 µL of 2% aqueous solution of glutaraldehyde was coated on the bioanode to crosslink the enzyme. After that, the modified electrode was dipped in DDW for 2 minutes to wipe off the unloaded enzyme and kept the modified electrode in the refrigerator (4 °C) before conducting the experiment. The remaining electrodes were fabricated similarly.

## Results and discussion

### FTIR analysis

**Figure 1:**
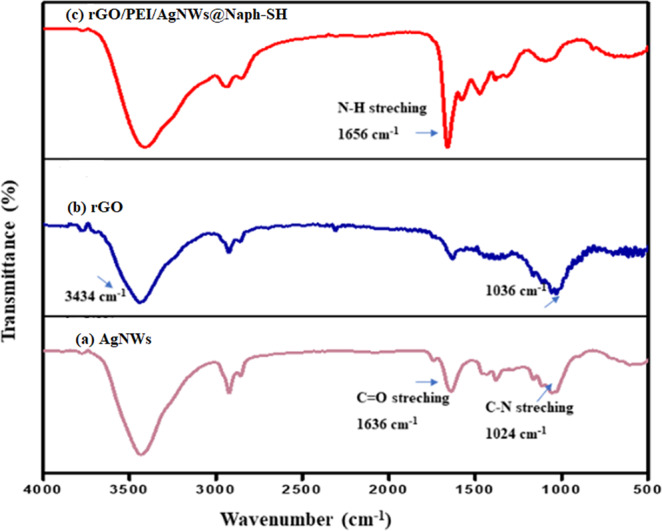
FTIR spectra of (**a**) AgNWs (**b**) rGO (**c**) rGO/PEI/AgNWs@Naph-SH nanocomposite.

Figure 1 represents the FTIR spectra of the AgNWs, rGO and the synthesized rGO/PEI/AgNWs nanocomposite. In Fig. 1(a), the band at 1024 cm^−1^ attributes to the C-N stretching vibration of PVP whereas the vibrations around 1636 cm^−1^ indicate the presence of the C = O group in PVP^44^. The bands (Fig. 1(b)) at around 1036 and 3434 cm^−1^ can be assigned to the C-C and O-H stretching vibrations of rGO, respectively^45^. The FTIR spectrum of rGO/PEI/AgNWs nanocomposite as shown in Fig. 1(c) displays almost the same vibration peaks as found in the individual spectrum of its components including a peak at 1656 cm^−1^ due to N-H bending vibrations of PEI^46,47^.

### XRD analysis

**Figure 2:**
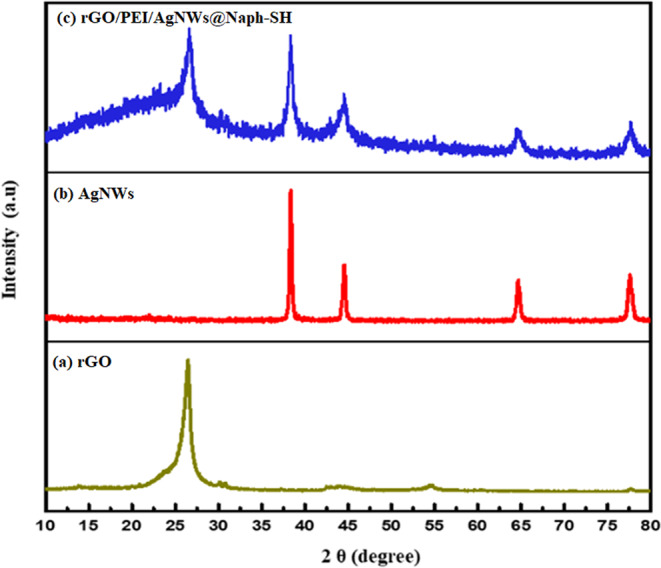
X-ray diffraction patterns of (**a**) rGO (**b**) AgNWs (**c**) rGO/PEI/AgNWs@Naph-SH.

The recorded X-ray diffraction patterns of the rGO and AgNWs were compared with as-prepared rGO/PEI/AgNWs nanocomposite as shown in Fig. 2. The vibrations corresponding to 26° can be attributed to the hexagonal plane (002) of rGO displayed in Fig. 2(a)^48^. The four sharp diffracting peaks of as-synthesized AgNWs in Fig. 2(b) were located at 2Ѳ angle of diffraction, for instance, 38.2°, 44.4°, 64.6° and 77.6° which are in agreement with the (111), (200), (220) and (311) diffractions of face-centered cubic (FCC)^42^. The diffraction peaks found in the synthesized rGO/PEI/AgNWs nanocomposite (Fig. 2c) represent almost all the diffraction peaks as found in each component and the amorphous nature appears due to the presence of PEI^49^. Hence, the XRD pattern also confirms the successful synthesis of the nanocomposite as well as of AgNWs.

### SEM and TEM analyses

**Figure 3:**
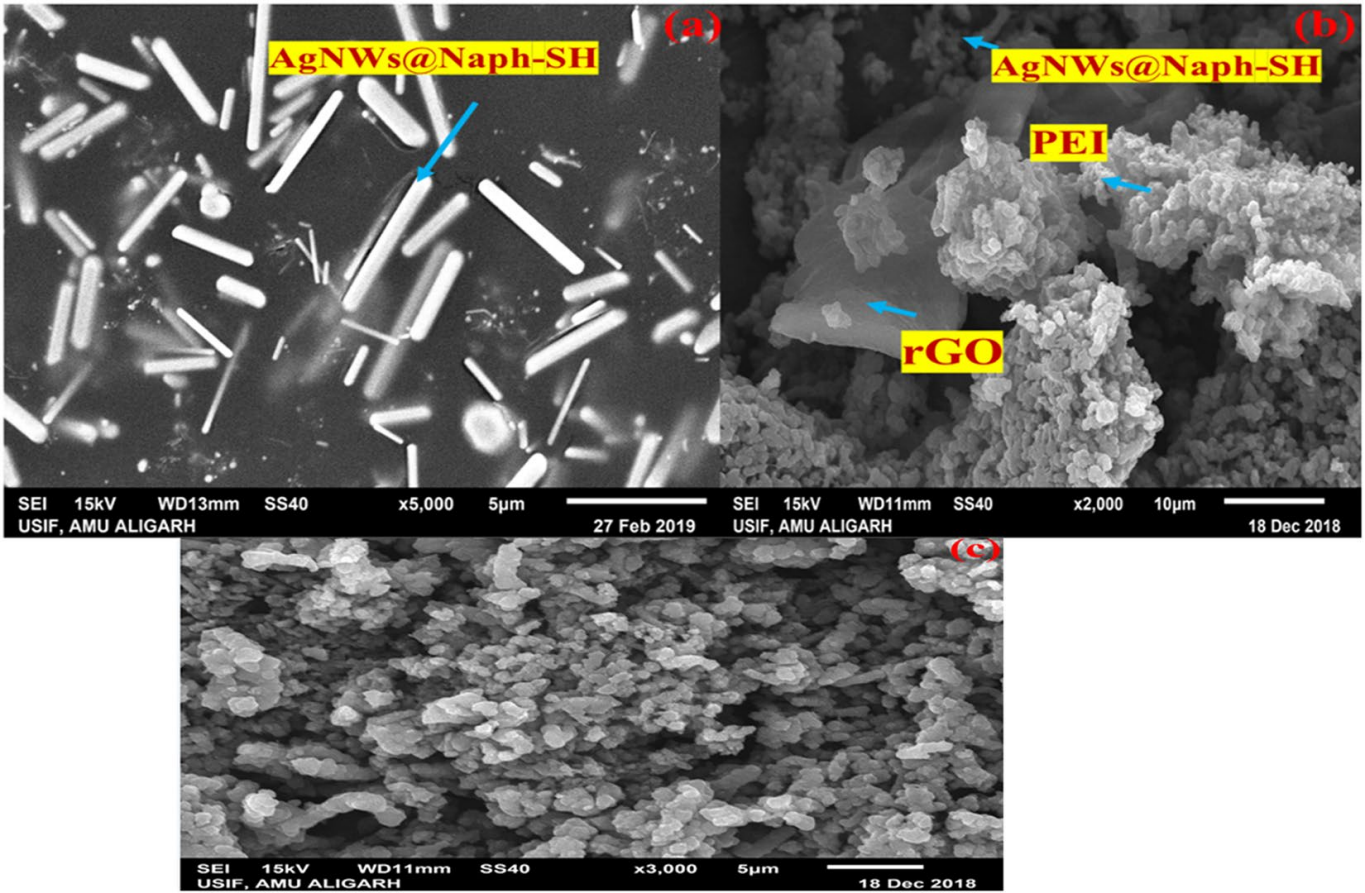
SEM micrographs of (**a**) AgNWs@Naph-SH (**b**) rGO/PEI/AgNWs@Naph-SH nanocomposite (**c**) rGO/PEI/AgNWs@Naph-SH/Frt/GOx bioanode.

**Figure 4:**
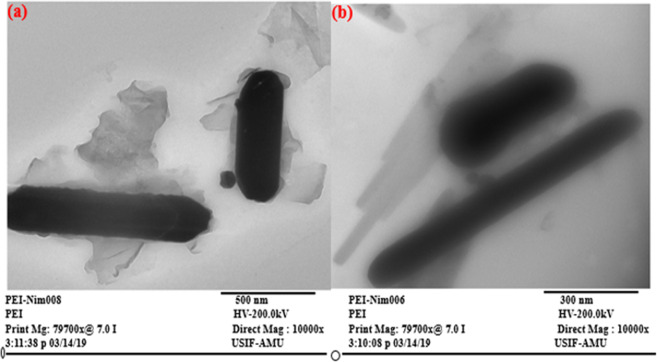
TEM micrographs of the rGO/PEI/AgNWs@Naph-SH nanocomposite (**a**) at 500 nm magnification, (**b**) at 300 nm magnification.

The morphology of the synthesized AgNWs and the nanocomposite (rGO/PEI/AgNWs@Naph-SH) are represented in the SEM micrographs. Figure 3(a) displays the rod shape, smooth texture and highly purified uniform nanowires of silver. From this image, it can be concluded that the AgNWs have been synthesized successfully. The SEM micrograph of nanocomposite illustrated in Fig. 3(b) clearly showed that the rGO sheets are wrapped around the PEI surface. The uniform dispersion of rGO sheets in the polymer matrix occurs due to the covalent interaction between rGO and PEI. Also, it can be seen in Fig. 3(c) that the rough surface ensures the wiring of GOx over the rGO/PEI/AgNWs@Naph-SH nanocomposite. Moreover, the TEM micrographs at different magnifications as shown in Fig. 4(a,b) revealed the non-uniform parallel arrays of AgNWs clinging to the surface of PEI and rGO sheets which further display the microenvironment of the nanocomposite. It could be concluded that AgNWs and rGO sheets act as fillers in a polymer matrix that provides a continuous path for electron transfer.

### Electrochemical studies

**Figure 5:**
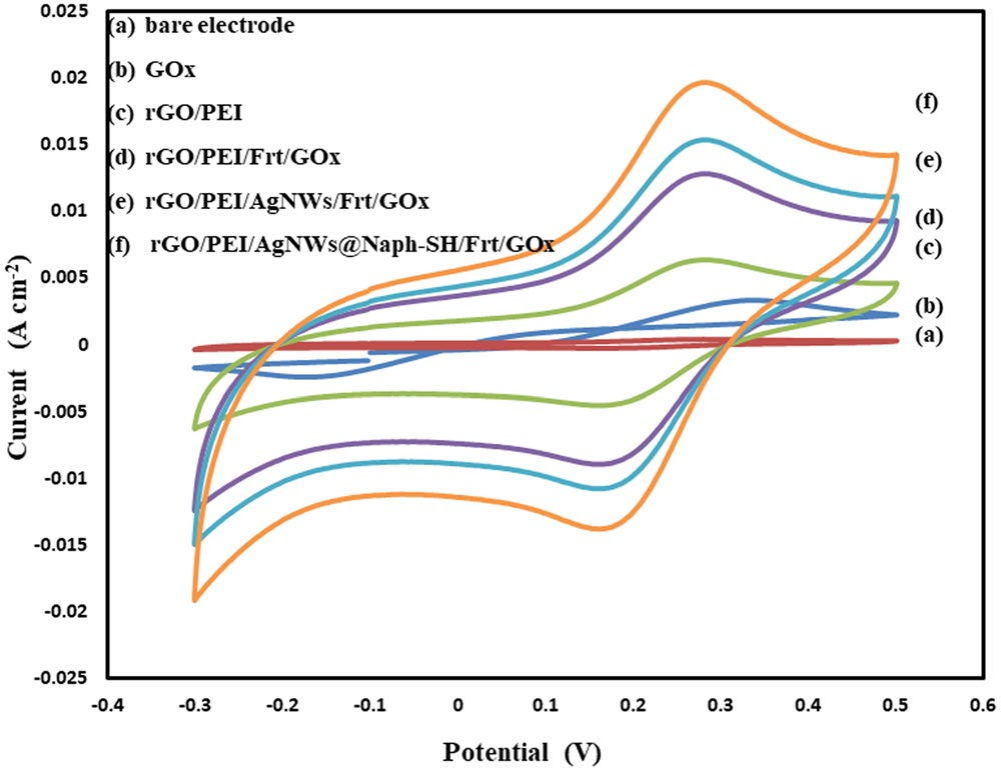
CVs of (**a**) bare electrode (**b**) GOx (**c**) rGO/PEI (**d**) rGO/PEI/Frt/GOx (**e**) rGO/PEI/AgNWs/Frt/GOx (**f**) rGO/PEI/AgNWs@Naph-SH/Frt/GOx bioanode in the presence of glucose (50 mM) in 1 M PBS (pH 7.0) as supporting electrolyte at an ambient temperature and a scan rate of 100 mVs^−1^.

The electrocatalytic performance of the modified electrodes namely, rGO/PEI/Frt/GOx, rGO/PEI/AgNWs/Frt/GOx, and rGO/PEI/AgNWs@Naph-SH/Frt/GOx were examined by cyclic voltammetry (CV). Before the examination, N_2_ gas was bubbled into the PBS (pH 7.0) to provide an inert environment. The electrochemical testing of the fabricated electrodes was conducted at the ambient temperature in 0.1 M PBS (pH 7.0) containing 50 mM glucose. In this study, the contribution of pure AgNWs and AgNWs@Naph-SH on the catalytic activity of biocatalyst was evaluated by comparing the CV curves of fabricated electrodes as shown in Fig. 5. The outcomes that appeared in CV curves illustrate that the current density obtained from the redox reaction of FAD (Flavin adenine dinucleotide) on the rGO/PEI/AgNWs/Frt/GOx bioanode was considerably higher than the rGO/PEI and rGO/PEI/Frt/GOx. It means that AgNWs favour the shuttling of the electron between the GOx and electrode surface, whereas the voltammogram of rGO/PEI/AgNWs@Naph-SH/Frt/GOx bioanode displayed further enhancement in the intensity of the catalytic current. This enhancement in the current density of rGO/PEI/AgNWs@Naph-SH/Frt/GOx bioanode can be attributed to the involvement of Naph-SH, which assists the electron shuttling by forming the Ag-thiol linkage. This linkage creates a hydrophobic interaction with the GOx that accelerating the electron kinetics of the reaction. Additionally, Naph-SH improved the electron transfer capability of PEI by promoting the electrical communication between the GOx and rGO sheets via π-π stacking^50^. Moreover, it can be seen from the figure that no catalytic activity was delivered by voltammogram of GOx due to leakage or poor immobilization of enzyme over the bare GCE.

**Figure 6:**
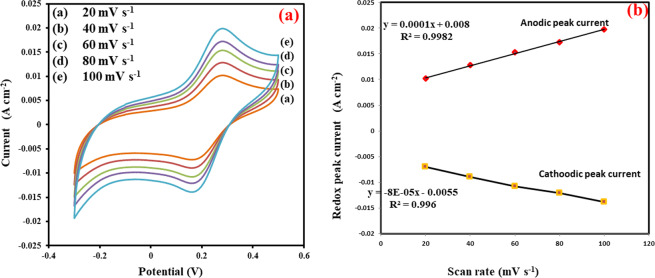
(**a**) CVs of rGO/PEI/AgNWs@Naph-SH/Frt/GOx at different scan rate ranging from 20–100 mV s^−1^ in presence of 50 mM glucose. (**b**) The respective calibration plot of redox peak current Vs scan rate (20–100 mVs^−1^).

Furthermore, the biocatalytic activity of the fabricated electrode rGO/PEI/AgNWs@Naph-SH/Frt/GOx was also studied as a function of scan rate ranging from 20 to 100 mVs^−1^ as shown in Fig. 6(a). From the figure, it is evident that by increasing the scan rate, the current density of the specified electrode increases linearly. The noticeable increment in current density is because of the high voltage applying that reduces the diffusion layer formed at the interface and facilitates electron transfer. The calibration plot of redox peak current Vs scan rate is given in Fig. 6(b). The curve demonstrates that the magnitude of peaks current (anodic and cathodic) rises as the potential scan rate increases linearly from 20 to 100 m Vs^−1^. So, it can be concluded that the catalytic reactions follow surface controlled processes^51^.

One of the valuable information which gives insight about the kinetics of the modified electrodes is the evaluation of rate constant (k_s_) using Laviron’s equation^52^. The k_s_ values of the three developed electrodes were evaluated as, 8.7 s^−1^, 9.8 s^−1^, 11.2 s^−1^ at 100 mVs^−1^ scan rate. More interestingly, the k_s_ values indicates that the use of AgNWs and AgNWs@Naph-SH contribute to enhancing the electron transfer rate. These outcomes show a good response as compared to the reported catalyst as shown in Table 1^53–58^.

**Table 1:** Comparative study of the heterogeneous rate constant (k_s_) of the bioanode with other reported work.

S.No.	Developed electrodes	k_s_ (s^−1^)	Reference
1.	rGO–PAMAM–Ag–GOD–CS	8.59	^53^
2.	RGO/Ag/GOx/GCE	5.27	^54^
3.	GOx-RGO/Nafion hybrid	3.78	^55^
4.	Kraton/MWCNTs/Frt/GOx	1.83	^56^
5.	rGO/PANI/f-Fe3O4/Frt/GOx	5.3	^57^
6.	ZnO/PIn-MWCNTs/Frt/GOx	4.28	^58^
7.	rGO/PEI/AgNWs@Naph-SH/Frt/GOx	11.2	Present work

Also, the surface concentration of the enzyme immobilized on rGO/PEI/AgNWs@Naph-SH/Frt/GOx was estimated to be 3.4 × 10^−7^ mol cm^−2^ by using Brown-Anson model as given in Eq. (1)^43^.

1 $${I}_{p}=\frac{{n}^{2}{F}^{2}{I}^{\ast }AV}{4RT}$$

where I_p_ = anodic peak current at 100 mVs^−1^ scan rate, I* = surface concentration of rGO/PEI/AgNWs@Naph-SH/Frt/GOx to be determined, A = surface area of GC electrode (0.07 cm^−2^). R (gas constants; 8.314JK^−1^), T (temperature, 298 K), F (Faraday’s constant; 96485 Cmol^−1^), n = 2 (number of electron transfer).

### EIS study

**Figure 7:**
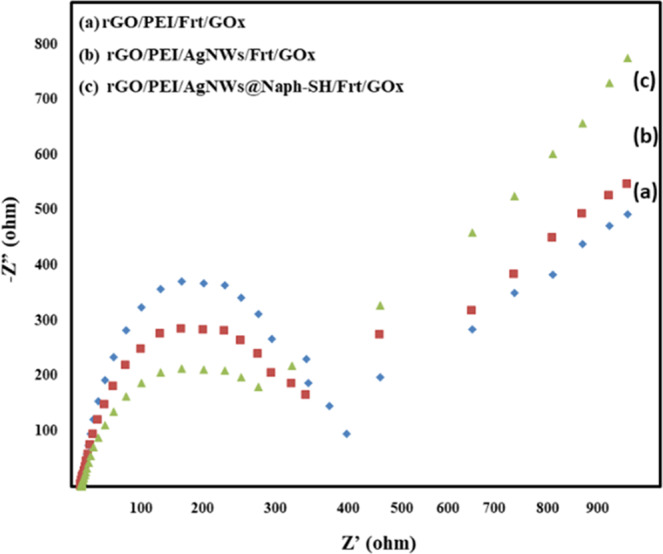
Nyquist plots of (**a**) rGO/PEI/Frt/GOx (**b**) rGO/PEI/AgNWs/Frt/GOx (**c**) rGO/PEI/AgNWs@Naph-SH/Frt/GOx in 50 mM glucose by using 1 M PBS (pH 7.0) solution at 100 mVs^−1^ scan rate.

Electrical impedance spectroscopy (EIS) was used to characterize the developed electrodes as shown in Fig. 7. By using Nyquist plots, the charge transfer resistance (R_ct_) of three fabricated electrodes was measured. Fig. 7 showed that the electrolyte resistance (Rs) remained the same for all the three developed catalysts. The rGO/PEI/Frt/GOx displays a well-defined semi-circle in the high-frequency region with the interface electron transfer resistance (R_ct_) of 430 Ω (curve c). After incorporating the AgNWs, the diameter of the semi-circle reduced corresponding to R_ct_ 410 Ω (curve b). The decrease in Rct ascribes the highly conducting nanowires of silver, which assist the electron shuttling by forming a continuous phase in the nanocomposite. Subsequently, the decrease in R_ct_ to 260 Ω (curve a) was obtained for rGO/PEI/AgNWs@Naph-SH/Frt/GOx. It can be due to Ag-thiol linkage which further enhances the flow of electron^50^.

### LSV analysis

**Figure 8:**
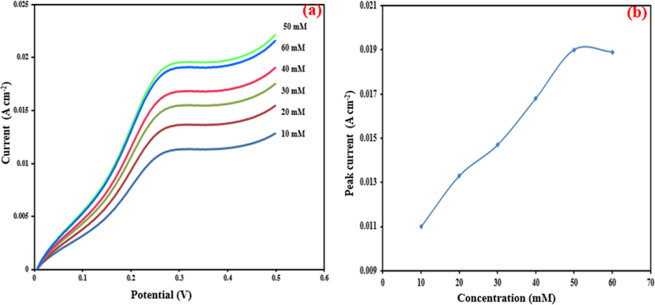
(**a**) LSVs of rGO/PEI/AgNWs@Naph-SH/Frt/GOx bioanode in 1 M PBS containing different concentrations of glucose varying from (10–60) mM at ambient temperature with a potential sweep rate of 100 mVs^−1^ and (**b**) The calibration curve corresponding to the electrolytic current against the variable concentration of glucose.

Linear sweep voltammetry (LSV) was carried out to optimize the current density by varying the glucose concentration of rGO/PEI/AgNWs@Naph-SH/Frt/GOx bioanode in PBS (pH 7.0). It is clear from Fig. 8(a) that the electrocatalytic current density enhances dramatically with the rise in glucose concentration from 10 to 50 mM. Upon addition of 60 mM glucose, there is a decrease in the anodic current, suggesting its saturation at 50 mM glucose. From this result, it can be concluded that the rise in current occurred due to the availability of vacant sites on the catalyst surface that are active to bind with substrate molecules and once all the active sites get filled, the saturation was reached leading to further decrease in current density. Moreover, the respective calibration curve of synthesized bioanode displays a maximum current density of 19.9 mAcm^−2^ at the 50 mM glucose concentration in Fig. 8(b). The proposed work is compared with the reported work as given in Table 2^18,56–59^. The current density of the proposed electrode is comparable with the electrodes already available. Moreover, the fabricated electrode can be used upto 45 days while keeping it into the refrigerator at 4 °C when not in use.

**Table 2:** Comparison of current densities of various modified electrodes with the present study.

Bioelectrode	Current density mA cm^−2^	Ref
Kraton/MWCNTs/Frt/GOx	1.14	^56^
rGO/PANI/f-Fe_3_O_4_/Frt/GOx	32.9	^57^
ZnO/PIn-MWCNTs/Frt/GOx	4.9	^58^
Ppy-Ag-GO/Frt/GOx	5.7	^59^
GCE/MnO_2_-G/PTA/Frt/GOx	3.68	
GC/MnO_2_-PSS-Gph/Frt/GOx	2.5	^18^
PEI/Naph-SH/AgNWs/Frt/GOx	19.9	Present study

## Conclusion

In this work, three nano-scaffold rGO/PEI, rGO/PEI/AgNWs, and rGO/PEI/AgNWs@Naph-SH were synthesized. The catalytic activity of these nano-scaffolds was tested by immobilizing enzyme (GOx) and mediator (Frt). The catalytic performance of the immobilized enzyme was examined by comparing the rGO/PEI/Frt/GOx, rGO/PEI/AgNWs/Frt/GOx and rGO/PEI/AgNWs@Naph-SH/Frt/GOx bioanodes. The maximum electrocatalytic current density, higher k_s_ 11.2 s^−1^ and lower charge transfer resistance (260 Ω) were found to be in the case of rGO/PEI/AgNWs@Naph-SH/Frt/GOx. Thus, our outcomes will likely open the doors for the development of more efficient EBFCs by using PEI-rGO films as the electrode supports.
